# Major Depletion of Plasmacytoid Dendritic Cells in HIV-2 Infection, an Attenuated Form of HIV Disease

**DOI:** 10.1371/journal.ppat.1000667

**Published:** 2009-11-20

**Authors:** Rita Cavaleiro, António P. Baptista, Rui S. Soares, Rita Tendeiro, Russell B. Foxall, Perpétua Gomes, Rui M. M. Victorino, Ana E. Sousa

**Affiliations:** 1 Unidade de Imunologia Clínica, Instituto de Medicina Molecular, Faculdade de Medicina, Universidade de Lisboa, Lisboa, Portugal; 2 Laboratório de Biologia Molecular, Serviço de Medicina Transfusional, Hospital Egas Moniz, Lisboa, Portugal; 3 Clínica Universitária de Medicina 2, Hospital de Santa Maria, Lisboa, Portugal; NIH/NIAID, United States of America

## Abstract

Plasmacytoid dendritic cells (pDC) provide an important link between innate and acquired immunity, mediating their action mainly through IFN-α production. pDC suppress HIV-1 replication, but there is increasing evidence suggesting they may also contribute to the increased levels of cell apoptosis and pan-immune activation associated with disease progression. Although having the same clinical spectrum, HIV-2 infection is characterized by a strikingly lower viremia and a much slower rate of CD4 decline and AIDS progression than HIV-1, irrespective of disease stage. We report here a similar marked reduction in circulating pDC levels in untreated HIV-1 and HIV-2 infections in association with CD4 depletion and T cell activation, in spite of the undetectable viremia found in the majority of HIV-2 patients. Moreover, the same overexpression of CD86 and PD-L1 on circulating pDC was found in both infections irrespective of disease stage or viremia status. Our observation that pDC depletion occurs in HIV-2 infected patients with undetectable viremia indicates that mechanisms other than direct viral infection determine the pDC depletion during persistent infections. However, viremia was associated with an impairment of IFN-α production on a per pDC basis upon TLR9 stimulation. These data support the possibility that diminished function *in vitro* may relate to prior activation by HIV virions *in vivo*, in agreement with our finding of higher expression levels of the IFN-α inducible gene, *MxA*, in HIV-1 than in HIV-2 individuals. Importantly, serum IFN-α levels were not elevated in HIV-2 infected individuals. In conclusion, our data in this unique natural model of “attenuated” HIV immunodeficiency contribute to the understanding of pDC biology in HIV/AIDS pathogenesis, showing that in the absence of detectable viremia a major depletion of circulating pDC in association with a relatively preserved IFN-α production does occur.

## Author Summary

Infection by HIV-2, the second AIDS-associated virus, is considered a unique natural model of attenuated HIV disease. HIV-2 infected individuals exhibit much lower levels of circulating virus (viremia) and progress to AIDS at slower rates than HIV-1 infected patients. In this study, we characterized for the first time blood plasmacytoid dendritic cells (pDC), important mediators between innate and acquired immunity, in HIV-2 infection. We observed a profound reduction in circulating pDC levels in HIV-2 infected patients, even in those with undetectable viremia, to levels similar to those found in HIV-1 infection. Moreover, we documented a more differentiated pDC phenotype in both infected cohorts relative to healthy individuals. Despite these similarities between HIV-1 and HIV-2 infections, pDC from HIV-2 patients with undetectable viremia exhibited, upon *in vitro* stimulation, a better-preserved ability to produce interferon-α (IFN-α), an important anti-viral cytokine with potential to stimulate other immune cells. Overall, our data suggest that the presence of virus in circulation, although not critical for the reduction in pDC number, appears to be central for the impairment of their function. This study of pDC in HIV-2 infection fills a gap in the understanding of their potential role in HIV/AIDS pathogenesis.

## Introduction

Plasmacytoid dendritic cells (pDC) are one of the two main subtypes of human dendritic cells. pDC, like the classical myeloid dendritic cells (mDC), are able to present antigens to T cells [1], but have a distinctive feature of producing type I interferons (IFN) [2]. pDC are able to secrete IFN-α at levels up to 1000 fold higher than any other blood cell following viral infection [2]. They recognize pathogens mainly via two pattern recognition receptors: Toll-like receptor 7 (TLR7), which recognizes single-strand RNA, and TLR9, which recognizes unmethylated DNA. The triggering of these receptors induces pDC activation and IFN-α production [3]. IFN-α is a potent stimulator of other immune cells, like mDC and NK cells, playing a central role in the development of immune responses, in addition to its well-documented antiviral effects [2].

pDC are thought to be particularly important in immune responses against viral infections, including HIV. Accordingly, IFN-α is one of the most important cytokines able to suppress HIV replication [4],[5]. However, increasing evidence suggests that IFN-α contributes to the generalized pan-immune activation and increased levels of cell apoptosis associated with AIDS progression, and thus the exact role of pDC in HIV/AIDS pathogenesis remains debatable [6]–[10].

HIV-2 infection is associated with low levels of circulating virus at all disease stages [11]–[15]. This is thought to be the main reason for the reduced HIV-2 transmission and its geographical confinement to West Africa and a few related European countries, in particular Portugal [16],[17]. Despite being associated with a clinical spectrum similar to HIV-1 [18], the rate of disease progression and CD4 decline is much slower irrespective of the disease stage [19],[20], leading to a limited impact on the survival of the majority of infected adults [21]. The reasons for the relatively benign course of HIV-2 infection remain poorly understood, and its potential to generate valuable insights into HIV immunopathogenesis has been little explored [16],[17],[22],[23]. Importantly, we have previously shown that in HIV-2 infected patients, as in HIV-1 infection, CD4 depletion is directly linked to immune activation [22],[24]. HIV-2 is closely related to HIV-1, sharing ∼60% homology at the amino acid level in the group antigens (GAG) and polymerase (POL) and 30–40% in the regions encoding the envelope protein (ENV) [23], and has been shown to be equally cytopathic *in vitro* [25]. Moreover, despite plasma viremia remaining low or undetectable throughout HIV-2 infection, the levels of proviral DNA do not significantly differ from those found in HIV-1 infected individuals [26]–[29]. These data suggest that HIV-2, like HIV-1, is able to disseminate and establishes a similar pool of infected cells. The reduced productive viral replication and the slow rate of the progressive immune activation and CD4 decline through the natural history of the disease are in agreement with distinct viral-host equilibrium during HIV-2 infection. Evidence exists to support preserved polyfunctional cellular specific responses [30]–[32], and broad neutralizing antibodies are found in HIV-2 infected patients [33],[34]. However, the debate continues as to whether these are the cause or the consequence of the control of viral replication and/or of a better preserved immune system [23]. Given the importance of the innate immunity in defining host-pathogen interactions, it is plausible that DC and other components of the innate response play a role. Accordingly, NK numbers and cytolytic activity have been shown to be better maintained in HIV-2 than in HIV-1 infection [35].

Importantly, a recent study showed that pDC are less susceptible to HIV-2 than to HIV-1 infection *in vitro* [36]. pDC express CD4 and both the HIV co-receptors CXCR4 and CCR5, and may be infected by HIV-1 *in vitro* and *in vivo* [37],[38]. Moreover, pDC apoptosis may be triggered by the binding of HIV-1 envelope in the absence of direct infection [39].

In this study we characterized for the first time circulating pDC in HIV-2 infected patients in order to generate insights into their role in HIV/AIDS pathogenesis. A similar marked reduction in the frequency of circulating pDC was found in untreated HIV-1 and HIV-2 infections that correlated with the degree of CD4 depletion and T cell activation, in spite of the absence of detectable viremia documented in the majority of HIV-2 patients. However, in contrast with HIV-1, IFN-α production upon TLR9 stimulation was relatively preserved in HIV-2 infection, except in the few HIV-2 patients with detectable viremia in whom major impairments were found.

## Results

### HIV-2 infection is associated with a marked reduction of circulating pDC that correlates with CD4 depletion

**Figure 1:**
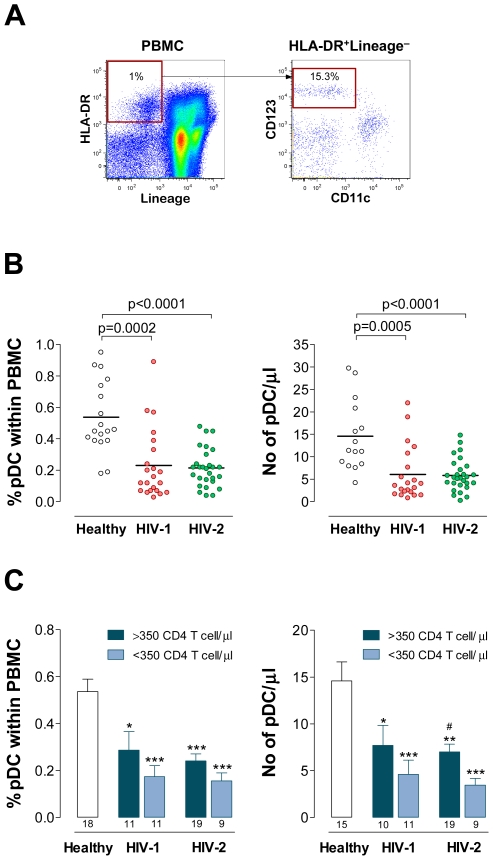
Similar reduction of circulating pDC in HIV-1 and HIV-2 infections. A representative flow cytometric pDC analysis is shown in (**A**). After a large gate including lymphocytes and monocytes defined by forward and side scatter, cells were further gated on HLA-DR^+^ cells that do not express lineage markers (left dot-plot). pDC were subsequently identified as CD123^+^CD11c^−^ cells (right dot-plot). Numbers represent the percentage of cells within the illustrated gates from an HIV-2 infected patient with 523 CD4 T cell/µl and undetectable viremia. The proportion of pDC within PBMC is 0.15% corresponding to 4 pDC/µl. (**B**) Graphs show circulating pDC levels in HIV-1 and HIV-2 infected cohorts and healthy controls expressed as percentage within PBMC and absolute numbers. Each dot represents one individual and bars represent mean. (**C**) The infected cohorts were stratified according to CD4 T cell counts (>350 and <350 cells/µl) and pDC levels compared as percentages and absolute numbers. Bars represent mean±SEM. Numbers under the bars represent the total individuals analyzed. ^*^p<0.05, ^**^p<0.01 and ^***^p<0.001 as compared to pDC levels in controls. ^#^p<0.05 between early and advanced HIV-2 cohorts.

HIV-2 infection is characterized by reduced to undetectable viremia [11]–[15] and a much slower rate of CD4 decline as compared to HIV-1 [19],[20]. We first asked whether this “favourable” outcome is associated with the preservation of circulating pDC. For this purpose, pDC were defined within freshly isolated peripheral blood mononuclear cells (PBMC) as HLA-DR^+^CD123^+^CD11c^−^ after the exclusion of cell-lineage markers, as illustrated in Fig. 1A. Cohorts of untreated HIV-2 and HIV-1 individuals with similar degrees of CD4 T cell depletion but distinct viremia were evaluated (Table 1).

**Table 1:** Characteristics of the cohorts studied.

	Healthy	HIV-1	HIV-2
Number (male/female)	18 (7/11)	22 (17/5)	28 (9/19)
Age, years	42±2	39±2^#^	48±3
	[25]–[57]	[23]–[61]	[19]–[78]
Ethnicity: Caucasian/Black	16/2	16/6	14/14
CD4 T cell count, cells/µl	935±63^a^	569±105^**^	666±80^*^
	[518–1397]	[18–1848]	[52–1511]
% CD4 T cells	44.9±2.0^a^	22.8±3.3^***^	28.5±2.6^***^
	[34.4–61.1]	[1.3–47.2]	[7.1–54.1]
% HLA-DR^+^ within CD4	4.1±0.4^b^	17.8±3.1^c; ***^	11.3±1.7^**^
	[1.9–7.6]	[1.7–54.5]	[1.9–36.3]
% HLA-DR^+^CD38^+^ within CD8	4.4±1.4^b^	29.7±4.2^c; ***^	20.6±3.6^***^
	[1.3–22.7]	[1.4–62.2]	[0.6–69.5]
Viremia, HIV RNA copies/ml	NA	672,310±294,026^d; ##^	3,035±1,125^d^
		[40–4,470,000]	[200–26,263]
Proviral DNA, copies/10^6^ PBMC	NA	172±55^e^	173±60^e^
		[5–975]	[5–1033]

Data are mean±SEM with limits in brackets. NA, not applicable. ^#^p<0.05 in comparison with HIV-2 infected patients; ^*^p<0.05, ^**^p<0.01, ^***^p<0.001 in comparison with healthy individuals.^ a^n = 17; ^b^n = 16; ^c^n = 21; ^d^4 out of 22 HIV-1 patients and 20 out of 28 HIV-2 patients had undetectable viremia. In these cases, the cut-off value of the test (40 and 200 RNA copies/ml for HIV-1 and HIV-2, respectively) was used to calculate the mean; ^e^Proviral DNA was quantified in 21 HIV-1 and 28 HIV-2 infected patients. It was detectable in all the patients evaluated but in 3 cases from the HIV-2 cohort it was below the cut-off value of the test (5 HIV DNA copies/10^6^ PBMC copies). In these cases, the cut-off value of the test was used to calculate the mean.

HIV-1 and HIV-2 infected patients exhibited a similar marked reduction in blood pDC as compared to seronegative controls, assessed both as percentage of total PBMC and as absolute numbers (Fig. 1B). This was not ascribed to sex, ethnicity or age distribution since no significant differences were found between males and females, Caucasians and non-Caucasians, and individuals with more or less than 45 years within each cohort or all cohorts combined (data not shown).

In order to evaluate whether the two infections also have similar levels of pDC depletion in early and advanced HIV disease, we stratified the HIV-1 and HIV-2 cohorts according to CD4 T cell counts (>350 and <350 CD4 T cells/µl). As previously reported [40]–[43], in HIV-1 infection pDC depletion was more marked in the advanced disease stage (Fig. 1C). Of note, we found comparable pDC levels in advanced HIV-2 infected patients (Fig. 1C). Moreover, a similar significant depletion was also documented in early disease in both infections as compared to seronegative subjects (Fig. 1C).

**Figure 2:**
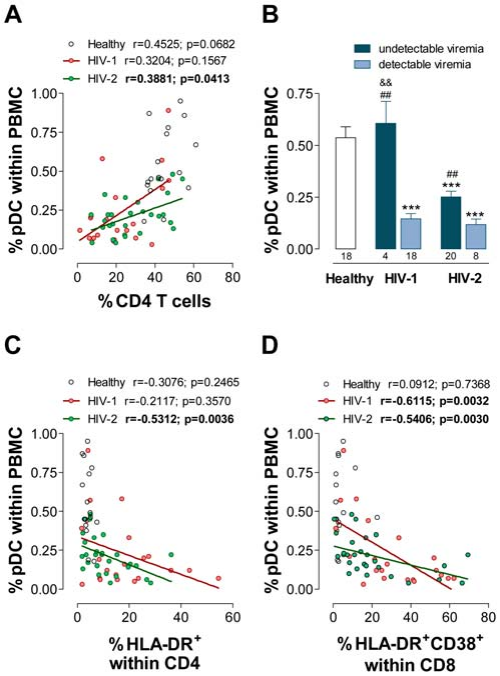
Relationship of pDC levels with CD4 T cell, T cell activation and viremia. Correlation between percentage of pDC within PBMC and percentage of CD4 T cells (**A**), percentage of HLA-DR within CD4 T cells (**C**), as well as percentage of HLA-DR^+^CD38^+^ within CD8 T cells (**D**), in HIV-2 and HIV-1 infections. Comparison of the pDC frequencies in the infected cohorts divided into viremic and “aviremic” (below test cut-off) groups (**B**). Bars represent mean±SEM. Numbers under the bars represent the total individuals analyzed. ^***^p<0.001 as compared to controls. ^##^p<0.01 between “aviremic” and viremic patients of the same cohort. ^&&^p<0.01 between HIV-1 and HIV-2 “aviremic” patients.

The association of pDC levels with disease progression in HIV-2 infection was further demonstrated by the statistically significant positive correlation found between the frequencies of pDC and circulating CD4 T cells (Fig. 2A). Although pDC levels were found to negatively correlate with the frequency of CD4 T cells in some HIV-1 studies [42]–[46], we found no significant correlation in our untreated HIV-1 cohort, possibly due to the reduced representation of patients with very low CD4 counts.

Hence, HIV-1 and HIV-2 diseases are associated with a similar extent of pDC depletion in spite of the slower rate of CD4 decline and the better prognosis that characterize HIV-2 infection.

### HIV-2 infected patients with undetectable viremia showed a marked decrease of circulating pDC

Plasma viremia is thought to be a major determinant of pDC depletion in HIV-1 infection [7], [40], [42]–[44],[47]. HIV-1 and HIV-2 infections are associated with markedly distinct plasma viral loads [11]–[15]. As shown in Table 1, 20 out of 28 HIV-2 infected patients had undetectable viremia and those with detectable viremia showed levels significantly lower than the ones found in HIV-1 patients. Of note, the highest viremia documented in the HIV-2 cohort was 26,263 RNA copies/ml.

In order to address the impact of viremia on the levels of circulating pDC, we divided the patients into two groups, viremic and “aviremic” (levels below the test cut-off). As shown in Fig. 2B, the HIV-2 group with undetectable viremia exhibited significantly lower pDC levels than the seronegative cohort. In addition, HIV-2 infected patients with detectable viremia had significantly lower pDC levels than the “aviremic” HIV-2 patients (Fig. 2B). However, it is important to stress that HIV-2 viremic individuals had significantly lower CD4 T cell counts than HIV-2 “aviremic” (356±60 cells/µl, n = 8, and 790±97 cells/µl, n = 20, respectively, p = 0.0112).

Nevertheless, a significant inverse correlation between the frequency of pDC within PBMC and viremia was observed in both HIV-2 (r = −0.4485; p = 0.0089; n = 28) and HIV-1 (r = −0.7684; p<0.0001; n = 22) cohorts.

As shown in Fig. 2B, HIV-1 individuals with undetectable viremia do not exhibit pDC depletion. The HIV-1 patients able to control viral replication in the absence of antiretroviral drugs are considered to represent less than 1% of HIV-1 infected individuals [48]. Our small group of 4 HIV-1 “aviremic” individuals had follow-ups with undetectable viremia ranging from 2 to 10 years (6.45±3.28 years of follow-up as compared to 7.03±1.21 in “aviremic” HIV-2) and showed relatively well preserved CD4 T cell counts (814±242 cells/µl, range 344–1425; as compared to 790±97 cells/µl, range 52–1511, in “aviremic” HIV-2). Similar findings were obtained when circulating pDC numbers were analyzed instead of pDC frequency (data not shown).

In summary, in agreement with previous reports [40],[42],[43],[47], we found a significant negative correlation between viremia and pDC levels in HIV-1 infection. However, a major reduction of circulating pDC levels was found in HIV-2 infected patients with undetectable viremia, showing that HIV-2 infected patients exhibited a major reduction in circulating pDC irrespective of the presence of detectable plasma viral load.

### The decrease in circulating pDC correlates with T cell activation in both infected cohorts

Both HIV-1 and HIV-2 infections are associated with a persistent generalized immune-activation, which is considered a main determinant of the immunodeficiency and that inversely correlates with CD4 T cell counts [22],[24],[49]. We assessed the relationship between pDC levels and expression of activation markers on T cells. HIV-2 infected cohort exhibited a significant inverse correlation between the frequency of pDC and the proportion of CD4 T cells expressing HLA-DR as well as of CD8 T cells that simultaneously expressed HLA-DR and CD38 (Fig. 2C and 2D). In the case of HIV-1 infection, a significant inverse correlation was only found with CD8 T cell activation, as shown in Fig. 2C and 2D. This is relevant since CD8 T cell activation is considered a better marker of the hyper-activation state associated with HIV infection with prognostic value [50]. On the other hand, CD4 T cell activation may be in part related to the homeostatic response to CD4 depletion, and, as described above, in our HIV-1 cohort no inverse correlation was documented between pDC and CD4 circulating levels. Similar findings were obtained in relation to the absolute number of circulating pDC as well as in relation to other parameters of CD8 T cell activation, namely the percentage and mean fluorescence intensity (FI) of CD38 expression (data not shown).

Overall, pDC depletion directly correlates with T cell activation in both infections.

### Phenotype of circulating pDC

**Figure 3:**
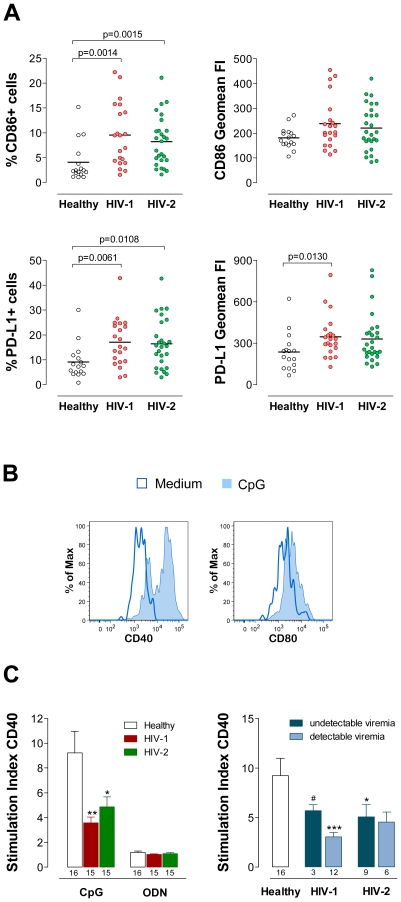
pDC Phenotype. (**A**) Phenotype of circulating pDC in HIV-1 and HIV-2 infections. Levels of expression of CD86 and PD-L1 on pDC from freshly isolated PBMC assessed as proportion within pDC and as geomean FI within total pDC measured by flow cytometry. Each dot represents one individual and bars represent mean. (**B**) pDC phenotype upon CpG stimulation. Freshly isolated PBMC were cultured with medium alone, CpG or the non-CpG ODN control. pDC phenotype was assessed by flow cytometry after 22 hours. Histograms represent the analysis of CD40 and CD80 expression upon CpG stimulation within pDC in a representative HIV-2 infected patient with 508 CD4 T cells/µl and 13627 HIV-2 RNA copies/ml. After a large gate including lymphocytes and monocytes defined by forward and side scatter, cells were sequentially gated on HLA-DR^+^ cells that do not express lineage markers and on CD123^+^CD11c^−^ cells. (**C**) The up-regulation of CD40 expression on pDC is showed as stimulation index defined as ratio between the geomean FI in the presence of CpG or ODN and in its absence (medium). Data are presented within total HIV-1 and HIV-2 cohorts (left graph) and within the infected cohorts grouped according to viremia status (right graph). Bars represent mean±SEM. Numbers under the bars represent the total HIV-1 and HIV-2 infected individuals as well as healthy controls analyzed. The subgroups of patients analyzed are representative of their respective patient population described in Table 1 with respect to CD4 counts and viral load. There are no significant differences between HIV-1 and HIV-2 cohorts. ^*^p<0.05, ^**^p<0.01 and ^***^p<0.001 as compared to controls. ^#^p<0.05 between “aviremic” and viremic patients of the HIV-1 cohort.

We next asked whether the phenotype of circulating pDC differ in the two infections. The co-stimulatory molecule CD86 was similarly overexpressed on pDC in the HIV-1 and HIV-2 infected cohorts and this increase was statistically significant as compared to healthy controls (Fig. 3A). No significant correlation was found between the CD86 expression, as assessed by percentage or geomean FI, and percentage of CD4 T cell (r = −0.0699 for HIV-1; r = −0.08539 for HIV-2, in the case of CD86 geomean FI) or viremia (r = −0.01922 for HIV-1; r = 0.1975 for HIV-2, in the case of CD86 geomean FI) in both infections. Moreover, we also found no correlation with the different parameters of CD4 and CD8 T cell activation evaluated.

The *ex vivo* expression of the co-stimulatory molecules CD40 and CD80 within pDC was minimal in all individuals (data not shown).

Programmed death-1 (PD-1) signaling mediates an inhibitory pathway of T cell response and its overexpression is considered to contribute significantly to the impairment of specific T cell responses in HIV-1 infected individuals [51]. We compared the expression of PD-1 ligands on pDC and found a statistically significant increase in the percentage of PD-L1^+^ pDC in both infections as compared to healthy controls (Fig. 3A). In addition, the increase in the PD-L1 geomean FI within total pDC also reached statistical significance in HIV-1 infected individuals in comparison with healthy controls (Fig. 3A). Again, no significant correlation was found between PD-L1 expression and percentage of CD4 T cell (r = 0.1200 for HIV-1; r = −0.02762 for HIV-2, in the case of PD-L1 geomean FI), or viremia (r = −0.02476 for HIV-1; r = −0.2501 for HIV-2, in the case of PD-L1 geomean FI), or the T cell activation markers assessed in both infections. pDC expression of PD-L2 was minimal in all the three cohorts (data not shown).

In summary, both CD86 and PD-L1 were similarly up-regulated on pDC of both HIV-2 and HIV-1 cohorts, irrespective of disease stage.

### Modulation of pDC Phenotype by TLR9 stimulation *in vitro*

pDC are known to express TLR9, which binds to unmethylated CpG motifs, and to mature upon TLR9 signaling [3],[52]. Studies on the modulation of pDC phenotype *in vitro* have been scarce and mainly conducted in HIV-1 patients under antiretroviral therapy [53]. We assessed the modulation of pDC phenotype upon TLR9 stimulation by stimulating freshly isolated PBMC with a TLR9 ligand (CpG type A) or a non-CpG oligodeoxynucleotide (ODN) as a negative control. After 22 h, cells were harvested and analyzed within a pDC gate as described above.

CD86 and PD-L1 were found to be up-regulated by the control non-CpG ODN (data not shown), precluding their use to evaluate pDC maturation induced by CpG. Therefore, we focused our analysis on the CD40 and CD80 molecules that, although exhibiting reduced *ex vivo* expression, were specifically up-regulated upon CpG stimulation (Fig. 3B). Results are shown as stimulation index (SI) calculated as the ratio of the geomean FI measured in the presence of CpG and medium alone. The capacity of pDC to up-regulate CD40 after CpG stimulation was significantly decreased both in HIV-1 and in HIV-2 individuals relative to healthy controls (Fig. 3C). An impairment of CD80 up-regulation was also documented in both infections, though without reaching statistical significance in comparison with controls (CD80 SI: 2.4±0.3 for seronegatives, 1.9±0.1 for HIV-1, 1.9±0.1 for HIV-2).

The stimulation index for CD40 geomean FI was found to have a significant positive correlation with the percentage of CD4 T cells (r = 0.8451; p<0.0001) and a negative correlation with viremia (r = −0.7312; p = 0.002) in the HIV-1 cohort, but no significant correlations were found in the HIV-2 cohort (r = −0.065 with percentage of CD4 T cells; and r = 0.0846 with viremia). Moreover, in contrast to the HIV-1 cohort, similar levels were found when HIV-2 patients with more and less than 350 CD4 T cells/µl (data not shown) or with detectable and undetectable viremia were compared (Fig. 3C). These data suggest that the impairment in CD40 up-regulation upon CpG stimulation was present throughout HIV-2 disease and was not further aggravated in late stages.

Overall, the circulating pDC of HIV-infected individuals showed a reduced ability to further differentiate upon CpG-A stimulation as compared to seronegative controls.

### Assessment of IFN-α production upon TLR9 stimulation

IFN-α production is mainly triggered through TLR7 and TLR9 [3]. CpG-A has been shown to preferentially act on pDC [52] and was used here to assess pDC ability to secrete IFN-α upon TLR9 stimulation. Using single-cell assessment by flow cytometry, we further confirmed that in our experimental conditions the CpG-A used selectively induced IFN-α production by pDC (Fig. S1).

**Figure 4:**
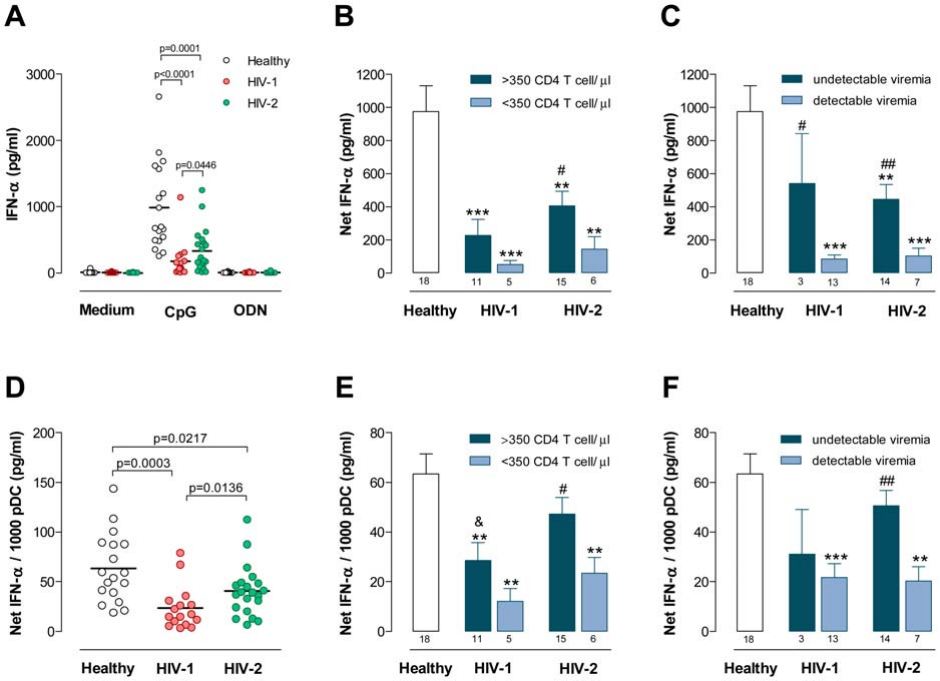
IFN-α production upon CpG stimulation. Freshly isolated PBMC were stimulated with CpG or the non-CpG ODN control. After 22 hours IFN-α was quantified in culture supernatants by ELISA. (**A**) Levels of IFN-α upon CpG or the non-CpG ODN control in healthy, HIV-1 and HIV-2 cohorts. The infected cohorts were further stratified according to CD4 T cell counts (**B**) or the presence or absence of detectable viremia (**C**) and the levels of IFN-α upon CpG stimulation are shown. Results expressed as “Net IFN-α” refer to the amount of IFN-α produced upon CpG stimulation subtracted by the IFN-α measured with medium alone. The “Net IFN-α” was divided by the total number of pDC in the culture and results are shown for the healthy, HIV-1 and HIV-2 cohorts (**D**), as well as for the infected cohorts stratified according to CD4 T cell counts (**E**) or the presence or absence of detectable viremia (**F**). Each dot represents one individual. Bars represent mean±SEM. Numbers under the bars represent the total individuals analyzed. The subgroups of patients analyzed are representative of their respective patient population described in Table 1 with respect to CD4 counts and viral load. ^**^p<0.01 and ^***^p<0.001 as compared to controls. ^#^p<0.05 and ^##^p<0.01 between the two groups of the same infected cohort. ^&^ p<0.05 between HIV-1 and HIV-2 patients with more than 350 CD4 T cells/µl.

Both HIV-1 and HIV-2 infected cohorts exhibited a significantly lower IFN-α production upon CpG stimulation as compared to healthy controls (Fig. 4A). Of note, similar levels of IFN-α production were found in the infected cohorts in Caucasians and non-Caucasians, individuals with more or less than 45 years, as well as males and females. Thus, despite recent data showing increased IFN-α production in women upon TLR7 stimulation *in vitro* [54], we were unable to detect any difference upon CpG stimulation.

**Figure 5:**
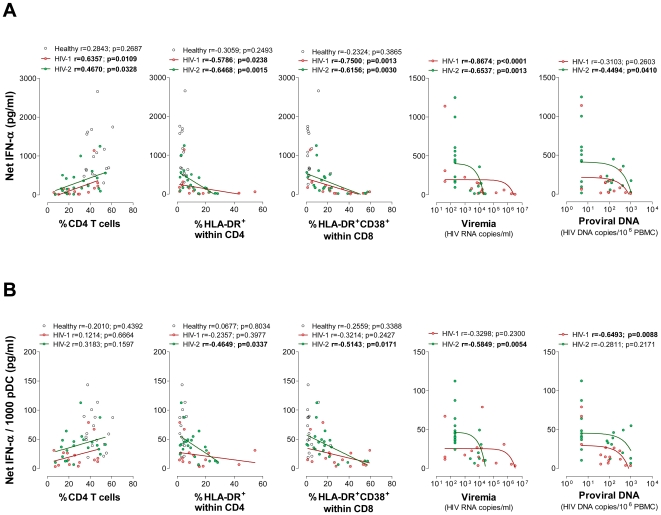
Relationship of IFN-α production upon CpG stimulation with CD4 T cells, T cell activation, viremia and proviral DNA levels. Correlations of frequency of circulating CD4 T cells, proportion of CD4 T cells that express HLA-DR, proportion of CD8 T cells that simultaneously express HLA-DR and CD38, viremia and levels of proviral DNA with the Net IFN-α production (**A**) and the Net IFN-α per 1000 pDC (**B**) upon *in vitro* CpG stimulation. Each dot represents one individual from the HIV-2 cohort, the HIV-1 cohort and healthy controls.

Patients with less than 350 CD4 T cells/µl tended to produce lower IFN-α levels than the patients with higher CD4 counts (Fig. 4B). In agreement, IFN-α production was found to positively correlate with the frequency of circulating CD4 T cells and inversely with the up-regulation of activation markers in CD4 and CD8 T cells in both infected cohorts (Fig. 5A). Noteworthy, the ability to produce IFN-α was also significantly lower in viremic than “aviremic” cohorts (Fig. 4C) and a significant correlation was found with viremia in both infections (Fig. 5A).

We estimated the IFN-α production on a per cell basis, by dividing the concentration of IFN-α produced upon CpG stimulation by the absolute number of pDC in the culture. Although there was a decrease in the ability of pDC to produce IFN-α in both infections, significantly higher estimated IFN-α levels per pDC were found in HIV-2 than in HIV-1 infected patients (Fig. 4D). Patients with less than 350 CD4 T cells/µl had lower levels of IFN-α production per pDC (Fig. 4E), but no significant correlation was found between IFN-α production per pDC and the degree of CD4 depletion in either infection (Fig. 5B). In addition, no correlation was found between the estimated levels of IFN-α production per pDC and CD4 or CD8 T cell activation in the HIV-1 cohort, in contrast with the significant correlations found in HIV-2 infection (Fig. 5B).

Importantly, when the cohorts were divided according to the viremia status, the HIV-2 patients with undetectable viremia exhibited preserved levels of IFN-α production per pDC (Fig. 4F). Moreover, detectable HIV-2 viremia was associated with a significant decrease in the levels of IFN-α per pDC, reaching levels similar to the ones documented in HIV-1 viremic patients (Fig. 4F). In agreement, a significant correlation was documented between IFN-α production per pDC and viremia in the HIV-2 cohort that was not observed in the HIV-1 cohort (Fig. 5B). These data suggest a major role of plasma viral load, even at low levels such as those found in HIV-2 patients, in the impairment of IFN-α production. Therefore, we next assessed the relationship between IFN-α production and proviral DNA. The levels of proviral DNA were similar in the HIV-1 and HIV-2 cohorts, including in “aviremic” groups (Fig. S2). No significant correlations were found between proviral DNA and pDC levels in both infections (data not shown). Of note, a significant correlation between viremia and proviral DNA was only found in the HIV-2 cohort (r = 0.4480, p = 0.0168 for HIV-2; and r = 0.0145, p = 0.9518 for HIV-1). Remarkably, a statistically significant positive correlation between proviral DNA and Net IFN-α production was found in the HIV-2 cohort that was not documented in HIV-1+ patients, showing that the higher the number of infected cells the higher the IFN-α production in HIV-2 infection (Fig. 5A). In contrast, the estimated levels of IFN-α per pDC correlated negatively with proviral DNA in the HIV-1 but not in the HIV-2 cohort (Fig. 5B). These data suggest that the number of infected cells contributed more to the impairment of IFN-α production on a per pDC basis upon TLR9 stimulation in HIV-1 than in HIV-2 infection, possibly related to the higher levels of effective viral replication in HIV-1 infection.

**Figure 6:**
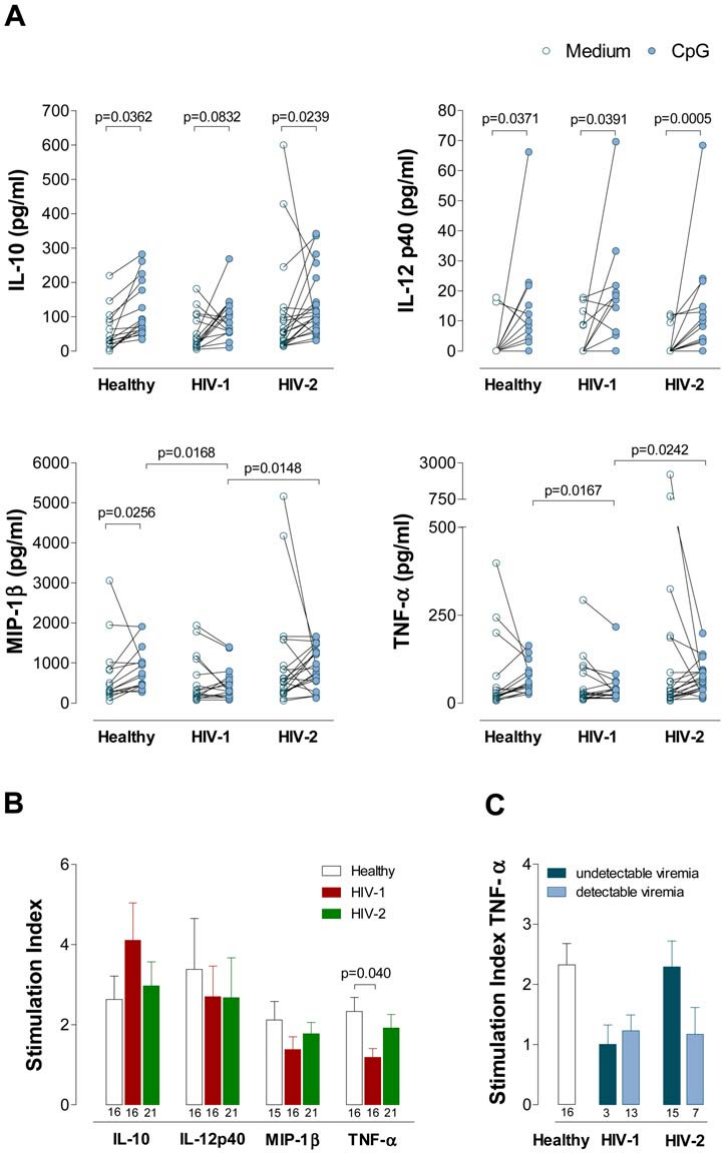
IL-10, IL-12p40, MIP-1β and TNF-α levels upon CpG stimulation in Healthy, HIV-1 and HIV-2 cohorts. Freshly isolated PBMC were cultured in the absence or in the presence of CpG. After 22 hours, culture supernatants were harvested and analyzed for the secretion of IL-10, IL-12p40, MIP-1β and TNF-α using the Luminex multiplex assay. (**A**) Cytokine levels. Each dot represents one individual. The levels observed in non-stimulated cultures (open symbols) are connected with those documented in the presence of CpG (closed symbols), and were compared using Wilcoxon test. (**B**) Stimulation indexes defined as ratio between the level of cytokine in the supernatant of the culture in the presence of CpG and in its absence (medium) in the three cohorts. (**C**) The HIV-2 and the HIV-1 cohorts were split according to the presence of detectable and undectable viremia and the stimulation indexes for TNF-α are shown. Bars represent mean±SEM. Numbers under the bars represent the total individuals analyzed. The subgroups of patients analyzed are representative of their respective patient population described in Table 1 with respect to CD4 counts and viral load. The Mann-Whitney test was used to compare the data of the HIV-2, HIV-1 and healthy cohorts, and the significant p values (<0.05) are shown.

CpG has been suggested to modulate the production of other cytokines both directly and indirectly through effects mediated by CpG-induced IFN-α [2]. We investigated the effect of CpG stimulation on the production of IL-10, IL-12p40, TNF-α and the β-chemokine MIP-1β by measuring their levels in the culture supernatants using a Luminex-based multiplex assay. Of note, the analysis of the combined cohorts revealed a direct correlation between the levels of IFN-α production upon CpG stimulation and the levels of TNF-α (r = 0.4033, p = 0.0027 for Net IFN-α and r = 0.4207, p = 0.0017 for Net IFN-α/1000pDC; n = 53) and MIP-1β (r = 0.3978, p = 0.0035 for Net IFN-α and r = 0.4272, p = 0.0016 for Net IFN-α/1000pDC; n = 52). No such correlations were found in the case of IL-10 or IL-12p40. As illustrated in Fig. 6A, the most striking finding was a reduced ability of HIV-1 infected individuals to produce MIP-1β and TNF-α, as compared to healthy and HIV-2 infected cohorts upon CpG stimulation. These decreases were clearer when the stimulation index was analyzed as illustrated in Fig. 6B, showing that a statistically significant reduction was only found in the case of TNF-α production in the HIV-1 cohort as compared to healthy controls. Of note, when the HIV-2 cohort was split accordingly to viremia status, the individuals with undetectable viremia exhibited a preserved ability to produce TNF-α (Fig. 6C) and MIP-1β (data not shown) in response to CpG stimulation, whereas the patients with detectable viremia showed a decrease in stimulation indexes similar to HIV-1 infected patients. However, despite the clear trends (p = 0.07 in the case of the viremic cohorts as compared to healthy controls), none of these differences reached statistical significance (Fig. 6C). These data suggest that viremia impacts on the ability to produce pro-inflammatory cytokines upon TLR9 stimulation *ex vivo* in HIV-2 infected individuals, as documented for IFN-α.

Despite the consensual data regarding the decreased ability of pDC to produce IFN-α upon *in vitro* stimulation in HIV-1 infected patients [40],[41],[47],[55], there are conflicting results regarding circulating IFN-α levels [43], [56]–[58]. To our knowledge, there are no reports of serum IFN-α levels in chronic HIV-2 infection. We assessed serum IFN-α by ELISA and found that, similarly to seronegative donors, all HIV-2 and the majority of HIV-1 infected patients had undetectable levels (<12,5 pg/ml). The only exceptions were two advanced HIV-1 infected patients with 13,18 pg/ml and 22,16 pg/ml of IFN-α (patients with 6.5 and 5.7 log10 RNA copies/ml, and with 18 and 290 CD4 T cells/µl, respectively).

In spite of the reduced circulating IFN-α levels and impaired production upon TLR9 and/or TLR7 stimulation *in vitro*, there is increasing evidence of increased IFN-α production *in vivo* during HIV-1 infection [7],[39],[59]. In order to evaluate this possibility, we quantified in freshly isolated PBMC the relative mRNA expression levels of *MxA*, a gene that is mainly induced by IFN-α and, thus, has been used as an indicator of IFN-α activity [46], [60]–[63]. As depicted in Table 2, significantly higher levels were found in HIV-1 than in HIV-2 infected patients. Importantly, in the HIV-1 cohort, the *MxA* levels were directly correlated with both viremia and levels of CD8 T cell activation, and inversely correlated with the frequency of circulating pDC and with the Net IFN-α production upon CpG stimulation *in vitro* (Table 2). No significant correlations were found in the HIV-2 cohort (Table 2). The “aviremic” patients tended to have lower levels of *MxA* expression than the viremic individuals in both infections, though not reaching statistical significance (relative *MxA* expression: 50±39 and 380±155, for the HIV-1 cohort; 81±23 and 122±50, for the HIV-2 cohort; respectively). These data are in agreement with lower activity of IFN-α *in vivo* during HIV-2 as compared to HIV-1 infection, that possibly explain the reduced refractoriness to IFN-α production upon further *in vitro* pDC stimulation observed in HIV-2 infected patients.

**Table 2:** Relative *MxA* mRNA expression.

	Healthy	HIV-1	HIV-2
Relative *MxA* mRNA expression	100±23	332±119^#^	92±20
Correlation of relative *MxA* mRNA with:			
% CD4 T cells	r = −0.2143	r = −0.3873	r = −0.2200
	p = 0.6191	p = 0.1246	p = 0.3132
Viremia, HIV RNA copies/ml	NA	**r = 0.6143**	r = 0.1260
		**p = 0.0087**	p = 0.5666
% HLA-DR^+^CD38^+^ within CD8	r = 0.1667	**r = 0.5172**	r = 0.2699
	p = 0.7033	**p = 0.0335**	p = 0.2130
% pDC within PBMC	r = 0.0952	**r = −0.6027**	r = −0.0763
	p = 0.8401	**p = 0.0104**	p = 0.7294
Net IFN-α, pg/ml	r = 0.1190	**r = −0.7011**	r = −0.1031
	p = 0.7930	**p = 0.0052**	p = 0.6938

Relative *MxA* mRNA expression was quantified by real-time PCR in 10 healthy subjects, 20 HIV-1 and 24 HIV-2 infected patients and expressed as mean±SEM of *MxA* mRNA copies/1000 GAPDH mRNA copies. Correlations were evaluated in 8 healthy, 17 HIV-1 and 23 HIV-2 individuals, except for Net IFN-α (14 HIV-1 and 17 HIV-2). ^#^p = 0.0421 as compared to HIV-2 cohort. NA, not applicable.

In summary, despite the similar decrease of pDC in both infections, pDC from HIV-2-infected patients appear to better preserve their ability to produce IFN-α upon CpG stimulation. Of note, no increase in the *ex vivo* levels of serum IFN-α was documented in HIV-2 infection in parallel with absence of evidence of significant increase in the IFN-α activity *in vivo*, as assessed by *MxA* relative expression levels. However, detectable viremia was associated with a similar impairment of IFN-α production in both infections, suggesting a major role of circulating virus in the pDC functional impairment *ex vivo*.

## Discussion

This study characterized for the first time circulating pDC in individuals with HIV-2 infection. A similar decrease in pDC levels was found in untreated HIV-2 and HIV-1 infections in spite of the much lower viremia and slower rate of disease progression that distinguishes HIV-2 disease. Importantly, a significant depletion of circulating pDC was documented even in HIV-2 infected individuals with undetectable viremia. The pDC levels were directly correlated with the degree of CD4 T cell depletion and T cell activation in both infections. Conversely, viremia appears to have a major impact on the ability of the remaining pDC to produce IFN-α upon TLR9 stimulation *in vitro* in HIV-2 infected patients.

HIV-1 infection has been consistently shown to be associated with reduced frequency and impaired function of circulating pDC, both during primary and chronic infection [40]–[44],[47],[55],[56]. These defects have been found to be more pronounced in individuals with higher viremia [40], [42]–[44],[47] and/or lower CD4^+^ T cell counts [42]–[44] and to be associated with the development of opportunistic infections and tumors [40]. Viral infection and induction of pDC apoptosis are thought to significantly contribute to the pDC depletion during both HIV-1 and SIV disease [39]. However, HIV-2 was shown to be less able than HIV-1 to infect pDC *in vitro* [36] and reduced levels of viral replication are documented in HIV-2 infected patients [11]–[15]. Therefore, the finding of a similar reduction of pDC levels was unexpected and suggests that it may be related to other mechanisms than direct viral effects. This possibility is further supported by the delayed and frequently incomplete recovery of pDC numbers and function following long-term successful antiretroviral treatment in HIV-1 seropositive patients [44],[47],[56]. Interestingly, the 4 HIV-1 infected patients with undetectable viremia in the absence of antiretroviral therapy evaluated in this study showed better preserved pDC levels than aviremic HIV-2 individuals. This was not apparently related to distinct length of patients' follow-up or proviral load, and could not be ascribed to the higher HIV-2 viremia cut-off, since viremia was quantified in the last 14 HIV-2 patients evaluated using an up-dated assay with a limit of detection of 40 RNA copies/ml and found to be undetectable (data not shown). The distinct pDC levels in the “aviremic” HIV-1 and HIV-2 groups suggest that the study of larger cohorts of these particular HIV-1 infected individuals, usually called “Elite controllers”, will be instrumental to better understand pDC biology in HIV/AIDS.

Traffic alterations have also been suggested to contribute to the reduced levels of circulating pDC. Several *in vitro* studies have documented a viral-associated up-regulation of molecules, such as CCR7 on pDC, that may contribute for their preferential homing to lymphoid tissues [64]. However, the changes in cell redistribution have not been consistently confirmed either in HIV-1 infected patients [7], [65]–[67] or in non-human primates infected with SIV [68]–[71]. Although there are studies that demonstrated an increase in pDC counts in lymph nodes and spleen during HIV-1 and SIV infections [65]–[67],[69],[70], other studies reported a parallel pDC decrease in the peripheral blood and lymphoid tissues [68] and an increase in pDC primed to apoptosis in the lymph nodes [71]. There are no data available on lymphoid tissues during HIV-2 disease. Of note, the establishment of HIV-2 infection is associated with levels of proviral DNA similar to those found in HIV-1, suggesting an equivalent viral dissemination despite the reduced HIV-2 viremia [26]–[29],[72]. Therefore, it is plausible that HIV-2 RNA and/or HIV-2 proteins may induce pDC maturation and migration. In agreement, we found the same alterations in the phenotype of circulating pDC in the two infections, and these changes were not associated with disease stage or viremia status. Of note, PD-L1 has been shown to be up-regulated in pDC upon TLR stimulation by HIV-1 products [73].

Strong correlations between pDC decline and up-regulation of markers of CD8 T cell activation both in HIV-2 and HIV-1 infections represent another important finding of our study. We have previously shown that generalized immune activation is likely to be a main determinant of HIV-2 disease progression [22],[24], as has been demonstrated in HIV-1 infection [22],[24],[50],[58]. Persistent HIV-2 infection is thought to induce a chronic stimulation of the immune system leading to a progressive T cell impairment and CD4 depletion, though at much slower rates than in HIV-1 infection [22],[24],[49]. The generalized pro-inflammatory state is likely to contribute to pDC depletion both by altered cell traffic and apoptosis susceptibility, as well as through the impairment of the ability of DC precursors to differentiate. We showed a similar up-regulation of the levels of expression of co-stimulatory and co-inhibitory molecules in the two infections, which may represent a state of incomplete differentiation that may preclude adequate antigenic presentation or be associated with tolerogenic properties, as previously reported [66],[74].

On the other hand, besides their antiviral properties, pDC are increasingly viewed as conductors of the immune activation associated with HIV/AIDS pathogenesis [6]. In this respect, a dual role has been suggested. A deleterious contribution of the pDC-mediated activation of other cells of the immune system [9] was further supported by the recently documented impairment of pDC activation and IFN-α production in sooty mangabeys, natural hosts of SIV infection that are known to exhibit reduced levels of immune activation and do not progress to AIDS [8]. Additionally, pDC were shown to be able to induce regulatory T cells upon activation by HIV-1 through an indoleamine 2,3-dioxygenase (IDO)-mediated mechanism [75] and, in this way, modulate immune activation and limit HIV specific responses [74],[75].

Despite some discrepant results, there is usually no significant increase in circulating IFN-α levels until advanced HIV-1 disease stages [43], [56]–[58]. In agreement, we were unable to detect increased serum IFN-α in the large majority of HIV-1 infected patients. We also found no detectable serum levels of IFN-α during HIV-2 infection.

Importantly, although a decrease in the pDC ability to produce IFN-α *in vitro* has been consistently observed during HIV-1 infection [40],[41],[47],[55], several lines of evidence suggest that there is increased production of IFN-α *in vivo*. HIV-1 infected patients have been shown to exhibit increased transcriptional levels of IFN-α and of several genes that are known to be induced by IFN-α [7],[39],[59]. There are no data on HIV-2 infected patients. Here, we showed that HIV-2 as compared to HIV-1 infection is associated with lower levels of *MxA* expression, a gene induced by IFN-α [60],[61]. Our data support a lower IFN-α activity *in vivo* throughout the course of HIV-2 infection, possibly contributing to the slower progression of immune activation and consequently lower rate of CD4 decline that distinguishes the HIV-2 disease [19],[20],[22],[24].

Worth noting, we found that in HIV-1 infection the levels of *MxA* expression directly correlated with viremia and were inversely related to the circulating pDC levels and the amount of IFN-α production upon *in vitro* TLR9 stimulation. These data further support the possibility that continuous pDC stimulation by TLR ligands *in vivo* leads to a refractory state of the pDC that explains the apparent paradox between reduced *in vitro* production of IFN-α and indirect evidence of increased IFN-α production *in vivo* [7]. HIV-1 itself has been shown to modulate pDC function, particularly through the binding of viral RNA to TLR7 [76], as well as through the impairment of TLR9 signaling by the envelope protein gp120 [77]. The corresponding HIV-2 envelope protein, gp105, was shown to exhibit distinct impacts in several immunological systems [78]–[81], but there are no data on its effects on TLR9 signaling.

We tested here the IFN-α production upon TLR9 stimulation *in vitro* and found it to be significantly impaired in HIV-2 infected individuals. Given the limited volume of patient samples, we selected CpG type A as a standard TLR9 ligand thought to target mainly pDC [52], as confirmed here by single-cell analysis of IFN-α production. A similar decrease in IFN-α production upon CpG stimulation of whole blood cultures was also recently reported in HIV-2 and HIV-1 infected cohorts in Guinea-Bissau, West Africa, as compared to non-infected individuals [82].

As discussed above, HIV-2 infected individuals are thought to have reduced levels of viral replication and, therefore, it is expected that their pDC would be exposed to much less HIV-related molecules able to signal through TLR. Importantly, when we split the HIV-2 cohort according to viremia status, we found that the individuals with undetectable viremia exhibited a preserved IFN-α production on a per pDC basis. These data suggested that despite their reduced number, pDC function was preserved in HIV-2 infected patients without detectable circulating virus. In contrast, a similar impairment in IFN-α production was found in viremic HIV-2 and HIV-1 infected patients despite the average 2 log difference in the number of plasma viral RNA copy numbers. These results suggest that even low levels of circulating virus are sufficient to intrinsically impair IFN-α production by pDC or to induce a pDC refractory state that prevents their subsequent response to further TLR9 stimulation.

In conclusion, we reported here for the first time a major depletion of circulating pDC during HIV-2 infection, a unique natural model of “attenuated” HIV immunodeficiency. This decrease was observed early in disease and also in HIV-2 infected patients with undetectable viremia, suggesting that mechanisms other than pDC direct viral infection play major roles in their depletion during persistent infections. On the other hand, viremia was associated with an impairment of IFN-α production on a per pDC basis upon TLR9 stimulation, in agreement with the possibility that diminished function *in vitro* is likely a consequence of prior activation by HIV virions *in vivo*.

## Materials and Methods

### Ethics statement

The study was approved by the Ethical Board of the Faculty of Medicine, University of Lisbon. Subjects gave written informed consent to blood sampling and processing. In exceptional cases, related to cultural factors, oral informed consent was chosen by the patient and the assistant physician provided a written declaration of the permission obtained.

### Studied cohorts

A cross-sectional study was performed involving 28 HIV-2 and 22 HIV-1 infected patients without ongoing opportunistic infections or tumours, followed at Hospital de Santa Maria in Lisbon, Portugal. 18 HIV-seronegative age-matched control subjects were studied. Cohort characterization is summarized in Table 1.

### Cell isolation and culture

PBMC were isolated from heparinized blood immediately after venopuncture by Ficoll-Hypaque density gradient centrifugation. PBMC were cultured at 2×10^6^ cells/ml in 24-well plates in 1.5 ml of RPMI1640 supplemented with 100 U/ml penicillin/100 µg/ml streptomycin, 2 mM glutamine (all from Gibco-Invitrogen, Paisley, U.K.) and 10% human AB serum (Sigma-Aldrich, St Louis, MO) at 37°C with 5% CO_2_, in the absence or presence of 10 µg/ml class A CpG-ODN 2336 (5′-gggGA**CG**A**CG**T**CG**TGgggggg-3′) or the non-CpG-ODN 2243 control (5′-gggGGAGCATGCTGgggggg-3′) provided by Coley Pharmaceutical Group (Wellesley, MA). After 22 h, cells were harvested for phenotypic analysis and culture supernatants stored at −80°C for subsequent cytokine evaluation.

### Cell surface staining by flow cytometry

PBMC surface staining was performed as previously described [83], and analyzed for pDC frequency *ex vivo* with the following anti-human conjugated antibodies: FITC conjugated lineage (Lin) markers (CD3 and CD14 from Sanquin, Amsterdam, Netherlands; CD16 from BD Biosciences, San Jose, CA; and CD20 from eBioscience, San Diego, CA); HLA-DR PerCP (L243, BD Biosciences); CD123 PE-Cy7 (6H6, eBioscience), and CD11c APC (B-ly6, BD Biosciences). Analysis was done within a large gate including lymphocytes and monocytes, defined according to their forward/side scatter characteristics. pDC were defined as Lin^−^HLA-DR^+^CD123^+^CD11c^−^. Gated pDC were further analyzed using PE-conjugated mAbs against CD40 (5C3) and PD-L2 (MIH18) from eBioscience, CD80 (L307.4, BD Biosciences) and APC-conjugated mAbs against CD86 (FUN-1, BD Biosciences) and PD-L1 (MIH1, eBioscience). The same strategy was applied to evaluate the phenotype of pDC after *in vitro* culture of PBMC with CpG-ODN. At least 400,000 events were acquired within a lymphocyte+monocyte gate, using a CANTO flow cytometer (BD Biosciences), and analyzed using FlowJo (Tree Star, Inc, Ashland, OR). The absolute numbers of pDC/µl of blood were calculated by multiplying their representation by the sum of the absolute lymphocyte and monocyte counts obtained at the clinical laboratory. The expression of pDC surface markers was evaluated both in terms of percentage and of geomean FI.

### Quantification of cytokines

IFN-α was quantified in serum samples and in culture supernatants using the VeriKine™ Human IFN-Alpha Serum Sample ELISA and Human IFN-Alpha ELISA Kit, respectively (PBL InterferonSource, Piscataway, NJ), according to manufacturer's instructions. IFN-α production at the single cell level was assessed by flow cytometry in PBMC cultured for 18 h in the presence of Brefeldin A (last 16 h culture; 10 µg/ml; Sigma) by intracellular staining with the anti-human IFN-α (clone 225.C; eBioscience) after surface staining, using a previously described protocol [84]. IL-10, IL-12p40, MIP-1-β and TNF-α were quantified in the supernatants of PBMC cultured as described above using the Human Cytokine LINCOplex Kit (Millipore Corporation, Billerica, MA) and the Luminex LX100 (Luminex Corporation, Austin, TX) according to manifacturer's instructions. Samples were assayed in duplicate.

### mRNA extraction and assessment of *MxA* expression by real-time PCR

Freshly isolated PBMC (1–5×10^6^ cells) were immediately placed into RLT lysis buffer (Qiagen, Valência, CA) and stored at −80°C. Lysates were further homogenized by passage through QIAshredder columns (Qiagen). Polyadenylated mRNA was extracted using Oligotex Direct mRNA Mini kit (Qiagen). mRNA was reverse transcribed into cDNA using random hexamers and Superscript II Reverse Transcriptase Kit (all from Invitrogen). mRNA and cDNA concentrations were determined using a NanoDrop ND-10 spectrophotometer (NanoDrop technologies, Wilmington, DE). *MxA* expression was determined by Quantitative Real-Time PCR using AbiPrism 7000 SDS thermocycler (Applied Biosystems) using an optimized kit prepared by PrimerDesign Southampton, UK, with the following protocol: enzyme activation (95°C for 10 minutes), followed by 45 cycles of denaturation (95°C for 15 seconds) and annealing and data collection (60°C for 60 seconds). Each sample was quantified in duplicate using 1 µg of cDNA in a 20 µl PCR mixture volume containing 10 µl of Platinum Quantitative PCR SuperMix-UDG, 0,4 µl ROX Reference Dye 50X (all from Invitrogen), 300 nM of *MxA* primers/probe mix or 300 nM of GAPDH primers/probe mix (internal control), both from PrimerDesign. Absolute quantities of mRNA product were determined from a standard curve of serial dilutions of known quantities of each specific amplicon (Primer Design). Results are presented as number of copies of *MxA* mRNA per 1000 copies of GAPDH.

### Proviral DNA quantification

Proviral DNA was quantified by real-time PCR based assays that amplify highly conserved regions in HIV-1 and HIV-2 *gag* using protocols that we have previously described [29]. The detection limit of the assays was 5 DNA copies/10^6^ PBMC.

### Plasma viral load assessment

HIV-1 viremia was quantified by RT-PCR (detection threshold of 40 RNA copies/ml, Roche, Basel, Switzerland). HIV-2 viremia was quantified using a RT-PCR-based assay [15] with a detection limit of 200 RNA copies/ml. The cut-off values of the tests were considered for the purpose of the analysis in the cases where detection was below this level.

### Statistical analysis

Statistical analysis was performed using GraphPad Prism version 5.00 (GraphPad Software, San Diego, CA). The data are presented as arithmetic mean ± SEM and were compared using Mann-Whitney test and Wilcoxon matched pairs test as appropriate. Spearman's correlation coefficient was used to assess the correlation between two variables. *P*-values <0.05 were considered to be significant.

## Supporting Information

Figure S1 CpG-type A selectively stimulate pDC to produce IFN-α. PBMC were cultured for 18 h in the absence and presence of CpG-A or its control ODN. Brefeldin A was added for the last 16 h of culture. Cells were intracellularly stained for IFN-α after surface staining. (A) IFN-α production by pDC cultured with medium alone, CpG or ODN. Dot-plots show the analysis performed within cells gated according to forward-scatter and side-scatter in order to include lymphocytes and monocytes, and subsequently gated in lineage negative cells (CD19^−^CD14^−^CD56^−^CD3^−^) and HLA-DR+ cells. IFN-α was selectively produced by CD123^+^ cells only after CpG stimulation. (B) Dot-plots illustrate the absence of IFN-α production after CpG stimulation within lineage positive cells including monocytes, T cells, NK cells and B cells. (C) The absence of IFN-α production by B cells is further confirmed by the dot-plots showing the staining of IFN-α within CD19^+^ lymphocytes.
(0.70 MB TIF)

Figure S2 Proviral DNA levels. HIV-2 and HIV-1 proviral DNA was quantified by real-time PCR within total PBMC. Graph shows the results of the HIV-2 and HIV-1 cohorts split according to viremia status.
(0.51 MB TIF)
