# Supplementary figures and images for: Major Depletion of Plasmacytoid Dendritic Cells in HIV-2 Infection, an Attenuated Form of HIV Disease
Source: PLoS Pathog. 2009 Nov 20;5(11):e1000667. doi: 10.1371/journal.ppat.1000667 (PMC2773933; doi:10.1371/journal.ppat.1000667)

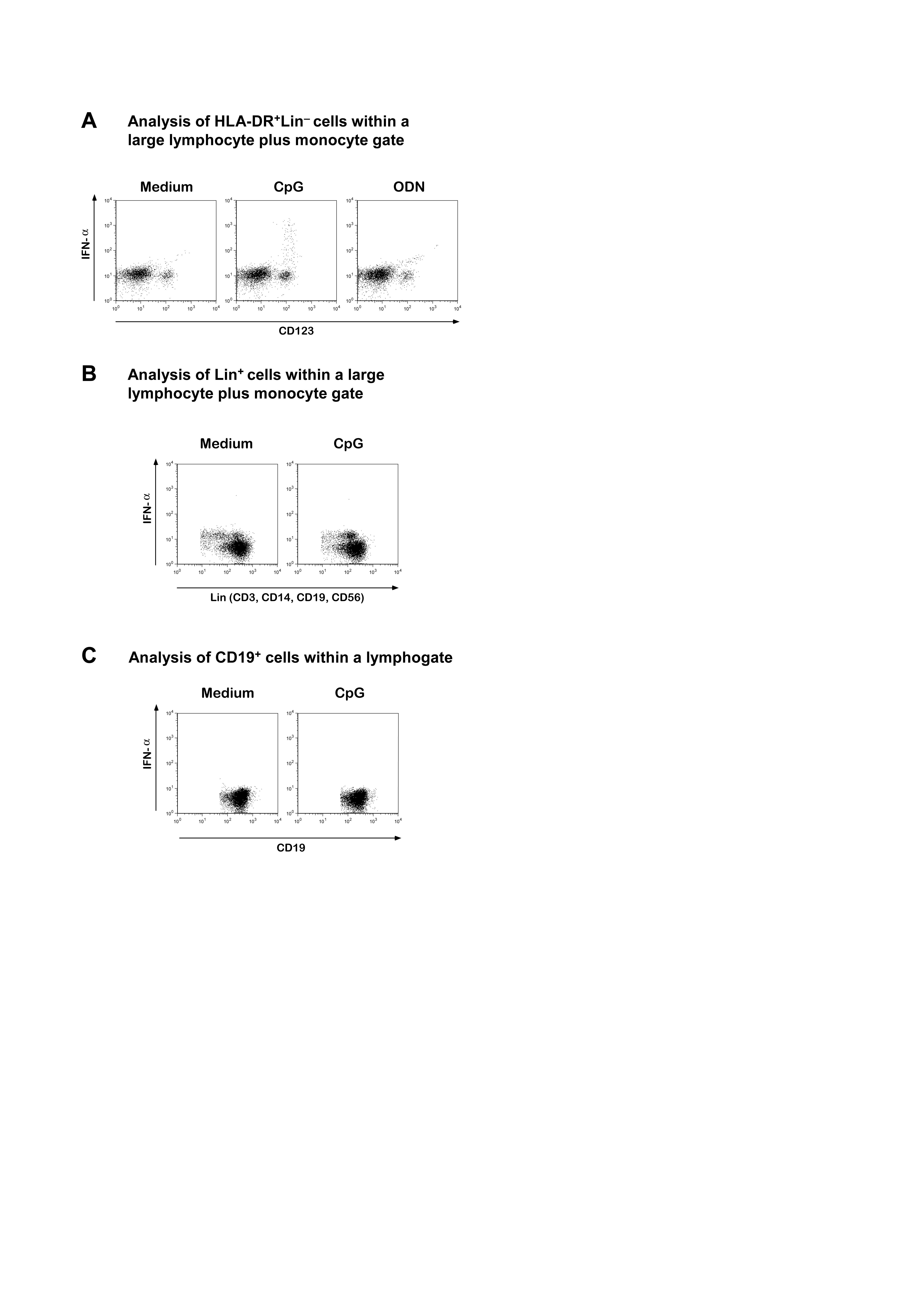

Supplement: Figure S1 — CpG-type A selectively stimulate pDC to produce IFN-α. PBMC were cultured for 18 h in the absence and presence of CpG-A or its control ODN. Brefeldin A was added for the last 16 h of culture. Cells were intracellularly stained for IFN-α after surface staining. (A) IFN-α production by pDC cultured with medium alone, CpG or ODN. Dot-plots show the analysis performed within cells gated according to forward-scatter and side-scatter in order to include lymphocytes and monocytes, and subsequently gated in lineage negative cells (CD19−CD14−CD56−CD3−) and HLA-DR+ cells. IFN-α was selectively produced by CD123+ cells only after CpG stimulation. (B) Dot-plots illustrate the absence of IFN-α production after CpG stimulation within lineage positive cells including monocytes, T cells, NK cells and B cells. (C) The absence of IFN-α production by B cells is further confirmed by the dot-plots showing the staining of IFN-α within CD19+ lymphocytes. (0.70 MB TIF) [file ppat.1000667.s001.tif]

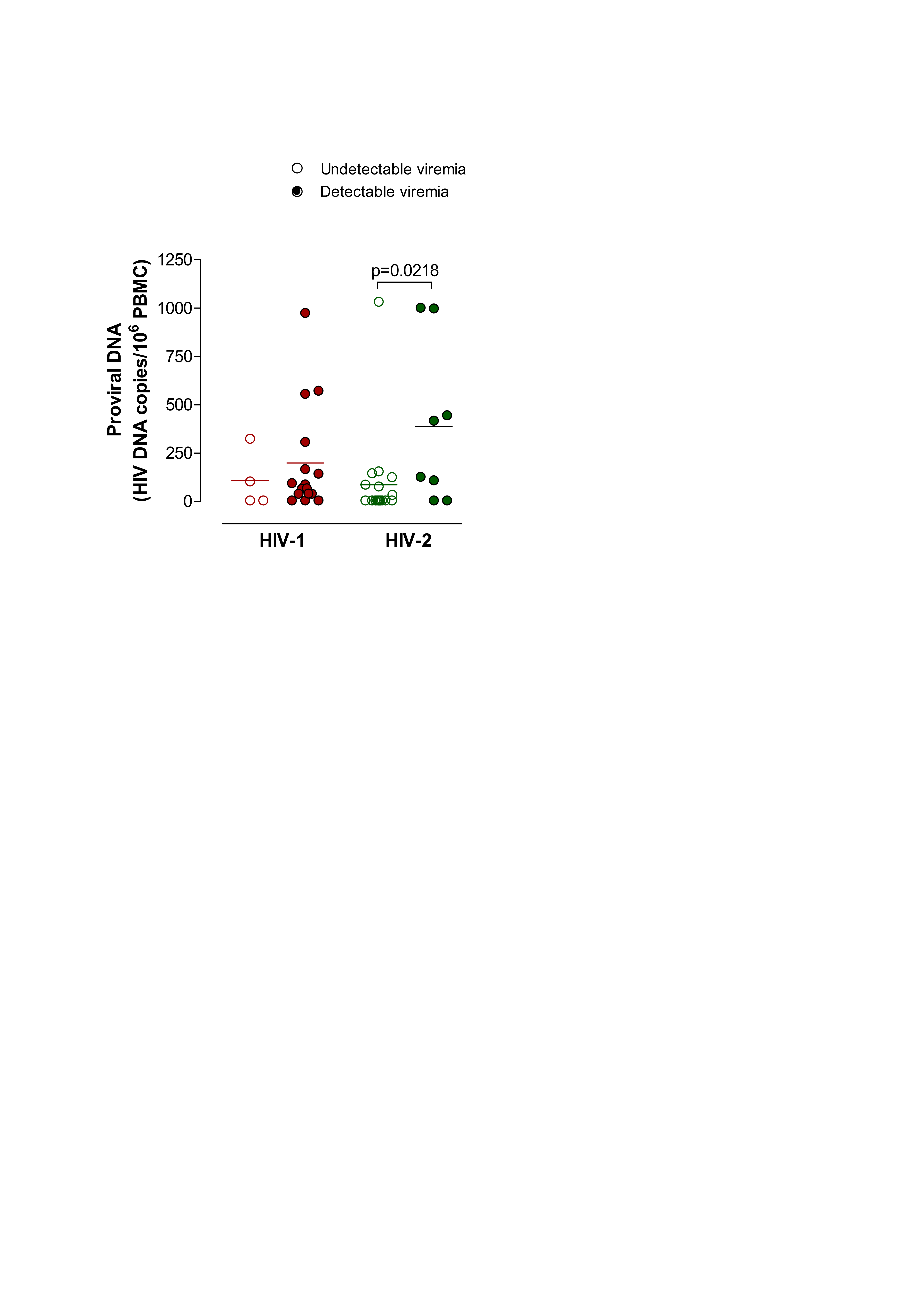

Supplement: Figure S2 — Proviral DNA levels. HIV-2 and HIV-1 proviral DNA was quantified by real-time PCR within total PBMC. Graph shows the results of the HIV-2 and HIV-1 cohorts split according to viremia status. (0.51 MB TIF) [file ppat.1000667.s002.tif]
